# Inhibition of inflammatory reactions in 2,4-Dinitrochlorobenzene induced Nc/Nga atopic dermatitis mice by non-thermal plasma

**DOI:** 10.1038/srep27376

**Published:** 2016-06-08

**Authors:** Jeong-Hae Choi, Yeon-Suk Song, Hae-June Lee, Jin-Woo Hong, Gyoo-Cheon Kim

**Affiliations:** 1 Department of Anatomy and Cell Biology, School of Dentistry, Pusan National University, Republic of Korea; 2 Department of Internal Medicine, School of Korean Medicine, Pusan National University, Republic of Korea; 3 Department of Electrical Engineering, Pusan National University, Republic of Korea.

## Abstract

Non-thermal plasma (NTP) has recently been introduced and reported as a novel tool with a range of medicinal and biological roles. Although many studies using NTP have been performed, none has investigated the direct relationship between NTP and immune responses yet. Especially, the effects of NTP on atopic dermatitis (AD) were not been explored. Here, NTP was tested whether it controls immune reactions of AD. NTP treatment was administered to pro-inflammatory cytokine-stimulated keratinocytes and DNCB (2,4-Dinitrochlorobenzene)-induced atopic dermatitis mice, then the immune reactions of cells and skin tissues were monitored. Cells treated with NTP showed decreased expression levels of CCL11, CCL13, and CCL17 along with down-regulation of NF-κB activity. Repeated administration of NTP to AD-induced mice reduced the numbers of mast cells and eosinophils, IgE, CCL17, IFNγ levels, and inhibited NF-κB activity in the skin lesion. Furthermore, combined treatment with NTP and 1% hydrocortisone cream significantly decreased the immune responses of AD than that with either of these two treatments individually. Overall, this study revealed that NTP significantly inhibits several immune reactions of AD by regulating NF-κB activity. Therefore, NTP could be useful to suppress the exaggerated immune reactions in severe skin inflammatory diseases such as AD.

Atopic dermatitis (AD) is a common chronic inflammatory skin disease associated with cutaneous hyper-reactivity to environmental triggers that are not harmful to normal individuals^1^,^2^. AD has a complex etiology featuring the activation of multiple immunologic and inflammatory pathways. About 80% of AD patients showed IgE-mediated sensitization and the remaining 20% are not IgE-mediated^3^, however, eosinophilia is associated with all AD patients. Many kinds of immune cells participate in the immunological responses of AD, which can be divided into two phases according to the chronicity of inflammation. The acute phase is mainly driven by T helper 2 (Th2) cells that actively secrete various cytokines including interleukin (IL)-4, -5 and -13^4^. These cytokines stimulate IgE production by B cells and the development of eosinophils^5^. Mast cells also play an important role as effector cells in IgE-mediated immediate hypersensitivity reactions^6^. On the other hand, in chronic phase AD skin lesions the immune response is dominated by Th1 cells^7^. After the acute immune responses, infiltrated and accumulated eosinophils and macrophages actively secrete IL-12, which stimulates the accumulation and development of IFNγ-expressing Th1 cells in the lesion. The increased level of IFNγ in the lesion exacerbates AD by stimulating the homing of immune cells and inducing spongiosis of the skin epidermis^8^. Not only immune cells but keratinocytes of the epidermis also communicate with the immune cells and actively participate in AD development by secreting several chemokines^9^.

There have been several methods used for treating AD; 1) the use of topical or systemic anti-inflammatory drugs^10^, 2) the enhancement of the skin barrier function using skin creams^11^, and 3) other ways including phototherapy^12^,^13^. Among these, the use of drugs for anti-inflammation is the most common and efficient prescription as a first-line therapy. However, a recent study reported that prolonged use of these drugs carries risks, because they can accumulate in the body and subsequently interfere with immune responses, so that other normal biological activities are perturbed^14^,^15^, Thus, the risk of adverse effects limits the use of these drugs. Meanwhile, skin barrier reconstructing creams are also often administered to AD patients as a defensive second-line therapy. Such creams can intensify the barrier system of the skin by adding lipid coats that form a defensive layer atop the epidermis^16^, but it has no or only a little anti-inflammatory activities. Phototherapy using UV-A or UV-B has quite recently begun to be used as an alternative treatment for AD^17^. Although the precise mechanism of UV’s curative effect on AD has not been elucidated, this method is known to inhibit the undesirable immune responses in AD skin lesions. However, UV treatment carries a high risk of genetic mutations, leading to various adverse effects including skin cancer^18^. Therefore, a safe and efficient technique to control the inflammation is strongly required for AD treatment.

In physics, the ‘plasma’ means the 4^th^ state of the matter, the highly active gas. Continuous technical research on plasma led to the development of non-thermal plasma (NTP), which has been used in biomedicine since the early 21st century^19^,^20^. NTP is believed to modulate various biological reactions because it contains many working elements including energetic electrons, ions, reactive oxygen species, etc^21^,^22^. Thus, many promising results have been yielded by research into NTP as a potential tool for sterilization^23^, cancer therapy^24^,^25^, wound healing^26^,^27^, tooth bleaching^28^ and anti-aging of skin^29^. However, there have been no reports concerning the direct effect of NTP on immune reactions in AD.

In this study, the possible role of NTP in regulating immune responses was explored. As a main target of NTP treatment of the skin, the HaCaT human keratinocyte cell line was used for *in vitro* experiments. To elucidate the effects and mechanisms of NTP as a modulator of keratinocyte-mediated immune reactions, NTP treatment was performed in the presence and absence of the pro-inflammatory cytokines TNFα and IFNγ, both of which play important roles in the development of AD. Furthermore, the effect of NTP on immune reactions *in vivo* was confirmed in DNCB-induced AD mice models, which were treated with NTP with or without co-treatment with 1% hydrocortisone cream (HC). Taken together, this study will highlight the NTP as a new tool for controlling cutaneous inflammation of AD.

## Results

### NTP treatment decreases the expression of CCL17 mRNA in HaCaT cells

**Figure 1:**
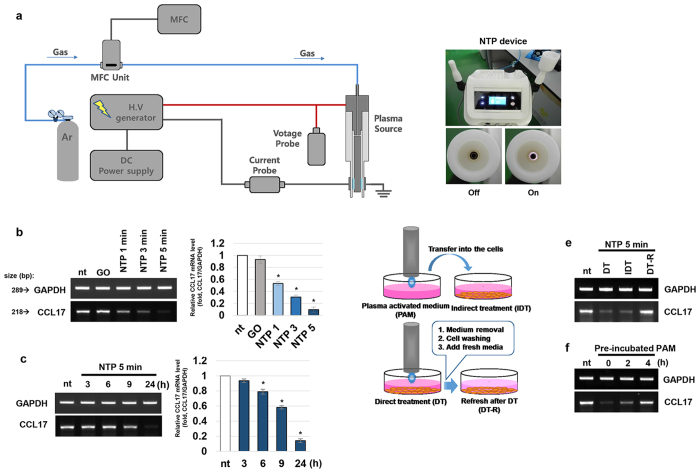
NTP inhibits the expression of CCL17 in keratinocytes. (**a**) A schematic diagram describing the NTP producing device (left panel) and the pictures of NTP device (right panel). (**b**) Treating time (plasma dose)-dependent effect of NTP on CCL17 expression. **GO**: gas only (argon gas), **nt:** non-treated cells. (**c**) Time-dependent CCL17 mRNA expression pattern after NTP treatment for 5 min. *p < 0.05 (**d**) A drawing describing the 3 different methods used for the treatment of HaCaT cells with NTP. (**e**) The differences among 3 NTP treatment methods in their effect on CCL17 expression by the cells. (**f**) The lifetime of NTP’s activity in influencing cellular CCL17 expression. Equal amounts of media (2 ml each) were treated with NTP for 5 min, then incubated in a cell culture chamber for 0, 2, and 4 h before being used for the treatment of cells. The data shown are representative of 3 separate experiments.

As a first step in this study, the effect of NTP on the expression of chemokine (C-C motif) ligand 17 (CCL17) in HaCaT cells was explored. As shown in Fig. 1a, HaCaT cells were treated with NTP in the presence of cell growth media. The expression of CCL17 was significantly decreased in a treatment time dependent manner, whereas the treatment of cells with vehicle gas (argon) had no effect on the expression of CCL17 (Fig. 1b). The effect of NTP on CCL17 expression was detectable from 6 h after the treatment (Fig. 1c). To achieve a better understanding of NTP -mediated CCL17 regulation, 2 alternative NTP treatment methods were used. In these methods, firstly, the growth media was refreshed after direct treatment to minimize the effects of chemical properties of NTP, and secondly indirect treatment was carried out to exclude the effects of physical properties of NTP on cells (Fig. 1d). When the cells were exposed plasma activated media (indirect treatment), the expression of CCL17 was inhibited, but the refreshment after direct treatment blocked the effect of NTP (Fig. 1e). To test the valid time of the NTP activity on CCL17 expression within the body, the pretreated media with NTP for 5 min were incubated in cell culture chamber for 0, 2, and 4 h without cells before the cell treatment. The effect of NTP pretreated media on CCL17 were remained about 55% after 2 h of pre-incubation, but only about 13% were shown after 4 h (Fig. 1f).

### NTP treatment inhibits the expression of CCL11, CCL13, and CCL17 in HaCaT cells

**Figure 2:**
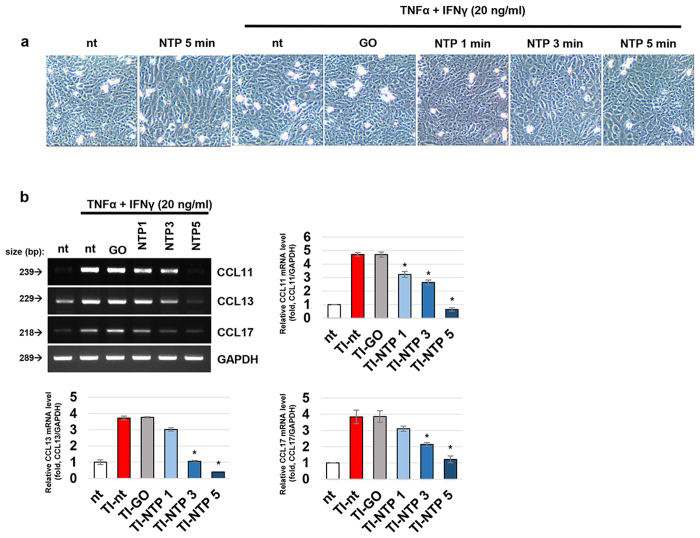
NTP inhibits TNFα and IFNγ-mediated immunological responses of HaCaT cells. The cells were treated with TNFα and IFNγ (20 ng/ml each) for 3 h, and then the cells were directly treated with argon gas (**GO**) or NTP for the indicated times. 24 h after the NTP treatment, the changes in the morphology and population density of the cells were monitored by taking a magnified picture of the cells (**a**), and then the cells were harvested and used for RT-PCR analysis (**b**). The data shown are representative of 4 independent experiments, *p < 0.05. **TI:** TNFα and IFNγ.

To trigger an AD-like immune response of in keratinocytes, TNFα and IFNγ were administered to HaCaT cells for 24 h. After treatment with these cytokines, HaCaT cells actively produced mRNA for immune cell homing chemokines CCL11, CCL13 and CCL17. While NTP treatment did not affect the viability or morphology of HaCaT cells (Fig. 2a), it reduced the expression level of CCL11, CCL13 and CCL17 in a treatment time-dependent manner (Fig. 2b). A significant decrease in the expression of all 3 genes was detected in the cells treated with NTP for more than 3 min.

### NTP treatment blocks NF-κB activation in an IκB independent manner

**Figure 3:**
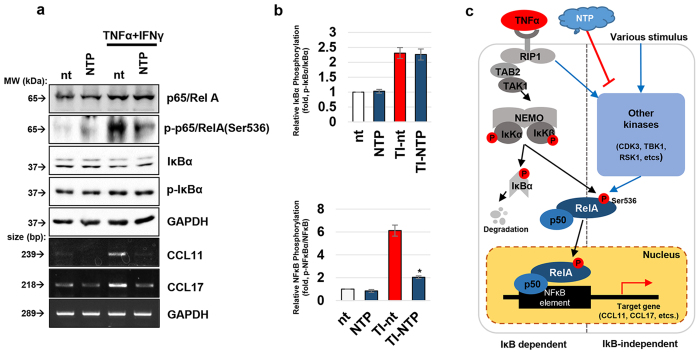
NTP treatment decreased NF-κB activity in an IκB-independent manner. (**a**) Duplicated set of cells were treated with TNFα and IFNγ (20 ng/ml each), NTP for 5 min as indicated. After 6 h and 24 h after the treatment, each set of cells were harvested and subjected to Western Blot and RT-PCR assay respectively. The data shown are representative of 4 independent experiments. (**b**) The relative activities of IκB and NF-κB were calculated by measuring the density of each band (total and phosphorylated IκB, p65/RelA) using the Image J analysis program (n = 4), *p < 0.05. **TI:** TNFα and IFNγ (**c)** A schematic diagram describing the NTP-mediated p65/RelA regulation.

Because NF-κB plays an important role in TNFα and IFNγ-mediated cell signaling, we investigated whether NF-κB activity was related to the NTP-mediated blockage of CCL11 and CCL17 expression. Western blot experiments showed that treatment of HaCaT cells with TNFα and IFNγ increased the expression of p65/RelA subunit of NF-κB and its phosphorylation at the Ser536 residue. Treatment with NTP significantly inhibited the increased phosphorylation of p65/RelA, whereas there was no effect on the total protein level (Fig. 3a). It is well known that phosphorylation at Ser536 of p65/RelA is important for the transcriptional activity of NF-κB^30^. Many kinases had been reported to participate in this phosphorylation such as IκK, CDK3, TBK1, RSK1, etc (Fig. 3c). In IκK dependent pathway, TNFα stimulates IκK complex which can phosphorylates IκBα along with p65/RelA^31^. Since the phosphorylation state of IκBα is important for the NF-κB activity, the effect of NTP on IκBα was also monitored. However, NTP treatment did not affect the phosphorylation of IκBα mediated by TNFα and IFNγ, although the phosphorylation of p65/RelA was decreased (Fig. 3a,b).

### Treatment of skin with NTP inhibits the recruitment of immune cells

**Figure 4:**
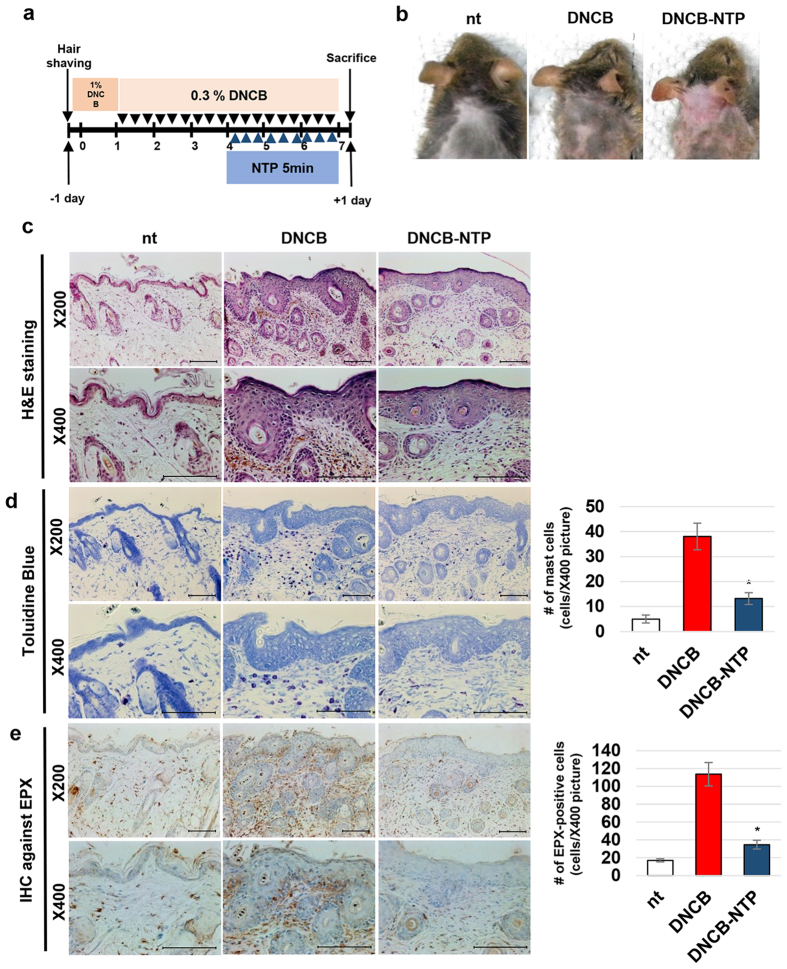
NTP treatment can block the accumulation of immune cells in DNCB-induced AD-like skin lesions. (**a**) A schematic diagram for the whole procedure of the *in vivo* experiments. (**b**) The morphological changes of mice at the end of the animal experiments. (**c**) The results of hematoxylin and eosin (H&E) staining. The pictures are representative of each group of mice (n = 5). (**d,e**) The results of toluidine blue staining and immunohistochemistry against eosinophil peroxidase (EPX). The Figures are representative of each group of mice (n = 5). The total number of each cell type in a picture taken at 400 × magnification were optically counted and averaged for each mouse using 5 randomly selected pictures, *p < 0.05. Scale bar: 100 μm.

In order to test the anti-inflammatory effect of NTP on AD, AD model mice were prepared by sensitizing Nc/Nga mice with DNCB and NTP were adopted as described in Fig. 4a. Treatment with DNCB for a total of 7 weeks induced wounds and pigmentation on the backs of the mice. However, the 9 times of NTP treatment on DNCB treated mice clearly reduced external phenotype of AD (Fig. 4b). Hematoxylin and eosin (H&E) staining showed that treatment of mice skin with DNCB resulted in an increased thickness of the epidermis and the recruitment of immune cells to the dermis (Fig. 4c). The treatment of NTP on these sensitized mice did not lead to any reduction in epidermal thickness, but the number of immune cells recruited to the dermis was reduced. In specific, DNCB sensitized mice showed 7.6 fold and 6.7 fold increase of mast cells and eosinophils respectively, but the NTP treatment on the sensitized mice reduced them into 2.9 and 1.9 fold (Fig. 4d,e).

### Treatment with NTP inhibits the DNCB-induced activation of NF-κB in skin

**Figure 5:**
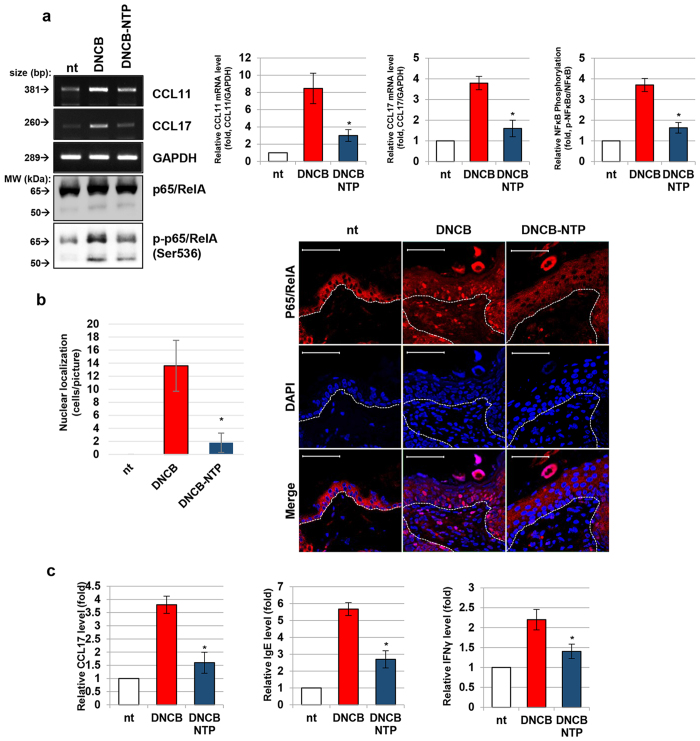
NTP treatment inhibits the DNCB-induced activation of NF-κB in the mouse skin tissues. (**a**) The results of RT-PCR and western blot analyses of mouse skin tissues. The data shown are representative of 4 independent experiments. (**b**) The results of an immunofluorescence (IF) assay against p65/RelA. The number of nuclei showing co-localization with p65/RelA staining were calculated by counting the nuclei stained in purple in the epidermis and averaged for each mouse using 5 randomly selected pictures. Scale Bar: 50 μm. **(c**) The results of ELISA analysis for the detection of CCL17, IgE, and IFNγ proteins in skin tissues. The data shown are representative of 4 independent experiments, *p < 0.05.

In order to confirm whether the inhibitory effect of NTP on DNCB-induced immune cell homing is related to the synthesis of chemokines, mRNA levels in skin tissues were analyzed. As shown in Fig. 5a, the level of CCL17 mRNA was increased by about 8.2-fold in the skin of mice treated with DNCB only, while in mice treated with DNCB and NTP, a lesser 2.5-fold induction of CCL17 was observed. Likewise, the DNCB-induced elevation of CCL11 mRNA expression (3.8-fold) was also reduced after NTP treatment (to 1.6-fold). Furthermore, western blot analysis of the skin tissues showed that the increased phosphorylation of p65/RelA at Ser536 by DNCB treatment was decreased after NTP treatment. The nuclear-localization of p65/RelA in DNCB sensitized mice skin were also completely blocked by NTP treatment (Fig. 5b). Finally, the DNCB mediated increase of AD marker proteins level (CCL17, IgE, IFNγ) were diminished with statistical significance after the NTP treatment (Fig. 5c).

### Combined treatment with NTP and hydrocortisone accelerates the healing procedures of AD mice

**Figure 6:**
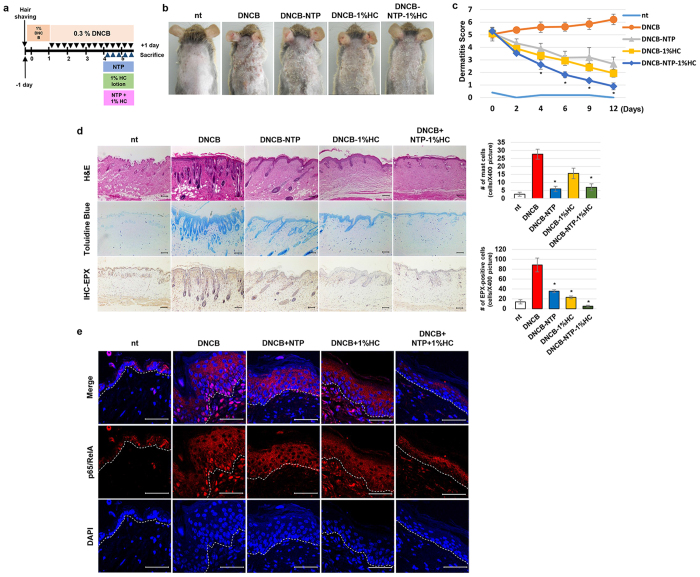
Combined treatment with NTP and 1% HC effectively reduces the healing duration of AD mice. (**a**) A schematic diagram describing the preparation of AD-like mice and the 3 different treatment methods used. (**b**) The morphological changes in mice after being treated 5 times with each method. The photographs are representative of each group of mice (n = 5), *p < 0.05. **(c)** The severity of external AD symptoms of the mice were measured by calculating dermatitis score at 0, 2, 4, 6, 9, 12 days after the 1^st^ application of NTP and 1% HC. *p < 0.05. (**d**) The results of H&E (top panel), toluidine blue (middle panel), and IHC against EPX (bottom panel). The photographs (X100) are representative of each group of mice (n = 5), scale bar: 100 μm. The total number of the EPX-positive or toluidine blue positive cells were in a picture taken at 400 × magnification were optically counted and averaged for each mouse using 5 randomly selected pictures, *p < 0.05. (**e**) The results of an IF assay against p65/RelA. DAPI was used for counter-staining the nuclei of cells in the skin tissues. Scale Bar: 50 μm.

For the best clinical results, medicinal devices can be used together with conventional drugs. To elucidate whether a combined treatment with NTP and 1% HC, which is one of the most frequently used drugs for treating AD, produces accelerated anti-inflammatory effects, a new set of *in vivo* experiments was performed as described in Fig. 6a. The treatment with either NTP or HC alone reduced DNCB-mediated wounds on dorsal skin, and the effect of HC was slightly stronger than that of NTP treatment. However, combined treatment with NTP and HC showed the greatest reduction in the severity of the DNCB-induced wounds (Fig. 6b). H&E staining (Fig. 6c) showed that treatment with neither NTP nor HP alone resulted in any reduction of the DNCB-induced thickening of the epidermis. However, combined treatment with NTP and HP strongly reduced the epidermal thickening. Five applications of treatment with NTP or HC cream led to a distinct reduction in the increased number of inflammatory cells induced by DNCB, and combined treatment with NTP and HC significantly blocked the DNCB-induced increase in the number of inflammatory cells. Toluidine blue staining showed that NTP treatment strongly inhibited the DNCB-induced recruitment of mast cells and that HC treatment moderately inhibited mast cell recruitment. On the other hand, HC inhibited the homing of eosinophils more efficiently than NTP. Combined treatment with NTP and HC demonstrated a superior effect in blocking the recruitment of mast cells and eosinophilic cells. The immunofluorescence assay for p65/RelA activity demonstrated that all 3 treatment methods inhibited the DNCB-induced activation of p65/RelA in the epidermis, but combined treatment with NTP and HC was more efficient in inhibiting p65/RelA activation in both the epidermis and dermis (Fig. 6d).

## Discussion

Recently, several studies have reported various beneficial properties of NTP that can be adopted for use in the field of dermatology. The application of NTP to wound healing is tested in clinical trials^32^, and a transdermal drug delivery approach using NTP was recently introduced as a safe and effective method^33^. NTP treatment was also reported to have anti-proliferative effects and stimulate apoptosis in melanoma cells^34^. The most well-known and broadly used medicinal property of NTP is its anti-bacterial and anti-fungal activity against various types of pathogens^35^,^36^. This function of NTP can be adopted for the treatment of skin diseases caused by microorganisms such as fungi and bacteria^37^. Isbary *et al.* reported the successful use of NTP in treating the lesions of Hailey-Hailey disease^38^, and Švarcová *et al.* reported a case in which NTP was used to treat superficial mycosis^39^. In both reports, the authors concluded that the strong anti-microbial activity of NTP greatly contributed to promoting the healing process. In several infectious skin diseases, the causative pathogenic microbes can trigger inflammation even after their death^40^,^41^. Furthermore, the exaggerated immune reactions in wound healing and infectious skin diseases typically cause a prolonged duration of healing^42^. Therefore, for the best treatment of wound healing and infectious skin diseases, both anti-microbial and anti-inflammatory activities are needed. To date, however, there have been no reports elucidating the direct effect of NTP on immune reactions.

This study demonstrates that NTP is an effective device for treating inflammation in skin lesions of AD. As a major source of CCL17 in the skin, keratinocytes actively participate in the exacerbation of immune responses within AD skin lesions. Saeki *et al.* proposed that CCL17 has an especially important role in AD as well as other skin diseases and that the expression pattern of CCL17 closely reflects the severity of AD^43^. Treatment with NTP effectively blocked the expression of CCL17 (Fig. 1a,c). Considering that treatment of cells with NTP-treated media inhibited the expression of CCL17, presumably various active chemical species generated from the plasma were incorporated into the media and then interacted with cells to repress CCL17 expression (Fig. 1e). UV-A is known to have an inhibitory effect on the expression of CCL17 in keratinocytes^44^, but in our results, the roles of UVs can be excluded for NTP mediated CCL17 inhibition. Since our NTP device generates ionized gas by adding electronic energy to argon gases on their path, the active chemical species of NTP can be perished easily. In our results, the anti-inflammatory activity of NTP had almost disappeared within 4 h in our *in vitro* system (Fig. 1f), suggesting that although NTP treatment might have side effects, it could interfere with other biological activities for short times. Thus, the potential of NTP to cause adverse reactions could be much less than that of drugs, which can remain in tissues for a long time.

During the development of AD, keratinocytes are stimulated by several kinds of cytokines that are secreted from immune cells, after which they actively participate in the process of AD by producing a broad spectrum of chemokines and growth factors. In this study, we showed that the treatment of keratinocytes with NTP effectively inhibited the TNFα and IFNγ induced expression of CCL11, CCL13, and CCL17 without causing cell damage (Fig. 2). These 3 chemokines are highly important for the induction of AD, because they stimulate the infiltration of the main effector cells such as eosinophils, mast cells, and Th2 cells into the lesions of AD. Meanwhile, the transcription factor NF-κB is a key protein complex involved in the regulation of various immune responses including the inducement of AD^45^. Due to the importance of NF-κB in several inflammatory skin diseases, many kinds of methods have been developed to inhibit NF-κB activity^46^,^47^,^48^,^49^,^50^,^51^,^52^,^53^. In this study, NTP treatment effectively inhibited the TNFα and IFNγ-induced activation of NF-κB by blocking the phosphorylation of its p65/RelA subunit at Ser536 (Fig. 3). Since NTP does not affect IκBα regulation, the NTP-mediated inhibition of NF-κB is surmised to be exerted through the IκK independent manner.

The possible role of NTP in treating AD was studied using DNCB-induced AD mice further. As our results shows the inflammation of mouse skin induced by DNCB was significantly reduced by NTP treatment (Fig. 4). DNCB treatment resulted in a thickening of the epidermal layer and induced the homing of numerous immune cells into the dermis. However, NTP treatment effectively decreased the number of cells migrating into the dermis, even though the thickening of the epidermis was not affected. The homing of immune cells into the lesion is a typical event in the AD development. Infiltrated mast cells secrete various cytokines to cause hypersensitization of the skin lesion in AD. Mast cells not only enhance keratinocyte-mediated cytokine secretion but also stimulate the maturation of Th2 cells. After the maturation and accumulation of Th2 cells in the AD lesion, Th2 cells actively participate in the activation and clonal expansion of IgE-secreting B-cells^49^,^50^, leading to the further activation of various types of IgE-recognizing immune cells, including mast cells^51^. The formation of this positive feedback loop between immune cells exacerbates the immune reactions of AD^52^. An increased number of eosinophils is a histological marker in AD skin biopsies, and this phenomenon is essential for the development of AD^53^,^54^. In this study, while high numbers of mast cells and eosinophils were observed in the AD tissues following DNCB treatment, NTP treatment significantly decreased the numbers of both cell types in the dermis. Therefore, a decrease in the number of mast cells and eosinophils in tissue leads to a moderate hypersensitization of the skin lesion, and thereby the severity of AD symptoms can be reduced. The relationship between NF-κB-mediated chemokine expression and the curative effect of NTP on AD were explored further. Our results from RT-PCR, western blot and immunofluorescence analyses of the skin tissues demonstrated that NTP treatment effectively inhibited the DNCB-mediated activation of p65/RelA, leading to an inhibition of CCL11 and CCL17 transcription in the skin tissues (Fig. 5). In concordance with our *in vitro* data, these *in vivo* data suggest that the suppression of p65/RelA activity in keratinocytes is a major contributor to NTP-mediated suppression of the immune reactions within AD lesions.

In our previous report, we reported that the NTP treatment on skin increased the absorption rate of external EGF through the skin^33^. Considering this transdermal drug delivery promoting property of NTP, we suggest that the pre-treatment of skin with NTP before the application of HC might bring grater curative results. In this study, NTP was more potent than 1% HC in inhibiting the DNCB-mediated accumulation of mast cells, whereas 1% HC was more effective in decreasing the eosinophils population in the lesion than NTP (Fig. 6c). The combined treatment induced a much greater reduction in the number of eosinophils in the dermis than did 1% HC treatment alone. The application of NTP or 1% HC alone did not reduce the DNCB-mediated epidermal thickening, but the thickened epidermis was recovered by the combined treatment. Although the working mechanisms of NTP and 1% HC might differ from each other, the combined treatment effectively inhibited p65/RelA activity in DNCB-treated skin, compared with each treatment given alone (Fig. 6d). Therefore, the use of NTP in combination with conventional anti-inflammatory drugs could be helpful for shortening the healing duration of AD and minimizing the amount of the drugs required.

To date, few medical devices have been developed for treating AD. Some devices that produce UV have been applied to the treatment of AD, but their usage has been limited due to the dangers of UV exposure. Here we introduce NTP as a new technique controlling cutaneous inflammation of AD for the first time. In particular, NTP can directly block NF-κB-mediated cytokine synthesis in keratinocytes and thereby inhibit downstream immune reactions. Although the acting time of NTP within the body is expected to be short, NTP effectively reduced inflammation in AD mice skin. Unlike UV-device, NTP is activated gas. Therefore it might be helpful for treating other atopic diseases in respiratory system. Taken together, NTP device can be a noble medical device which can control several immune reactions safely and effectively.

## Methods

### Reagents

All chemicals were purchased from Sigma-Aldrich (St. Louis, MO, USA) unless otherwise indicated. The bioactive IFNγ and TNFα cytokines were purchased from Santa-Cruz Biotechnology (Dallas, TX, USA).

### NTP device

For this study, a new type of low-frequency NTP device was developed (Fig. 1a). This NTP device has two of argon plasma ejecting modules, a module ejecting a single plasma flow for treating small areas (~30 mm^2^) and a module ejecting 3 plasma flows which can cover larger area (~60 mm^2^). The single plasma ejecting module is composed of ceramic body as a dielectric, and inner and outer electrodes. The outer electrode is grounded and a sinusoidal high voltage is applied to the inner electrodes by a high voltage source which can increase the voltage up to 10 kV with a frequency of 15 kHz. Argon gas was used for a buffer gas and blown into the plasma source with 2 slm (standard liter per minutes). When Vapp increases up to about 2.8 kV, the plasma starts to be generated between two electrodes. The plasma glow formed within the electrodes, but does not extended to the end of the electrodes. The temperature of the NTP flow at 1 cm from the electrode end was maintained under 35 °C for 10 minutes, and no UVs were detected at this condition. The single plasma module was used for treating cells, whereas the triple-plasma module was used for mouse experiments. The distance of treating subjects from the electrodes were kept as 1 cm.

### RT-PCR analysis

Total RNA was extracted using TRIzol^®^ reagent (Life Technologies, Carlsbad, CA, USA) for RT-PCR analysis. The detailed methods for RT-PCR analysis are described elsewhere^29^. The primer sequences used in this study are CCL11 (human: 5′-CCAATTCGATCCCCTGTCA-3, 5′-CCCCTCAGCTCAGTGTGG-3′, mouse: 5′-GGGGGGCATGAAAGGAGA-3′, 5′-TGGCTTGGCATGGTAGCAC-3′), CCL13 (5′-GGATGCATTCGGTTTTGTGA-3, 5′-CATGACTCCCACAGGCATG-3), CCL17 (human: 5′-AACTGTGCAGGGCAGGGCC-3′, 5′-TGTGGCTCTTCTTCGTCCCTG-3′, mouse: 5′-AGGGCAAGCTCATCTGTGC-3′ 5′-GGGAGGAAGGCTTTATTCCG-3′), GAPDH (5′-ACTGGCATGGCCTTCCGT-3′, 5′-CCACCCTGTTGCTGTAGCC-3′).

### Western blot analysis

Thirty micrograms of the protein lysate was resolved by SDS/PAGE (8–10% gel) and transferred to PVDF membranes (Millipore, Billerica, MA, USA). Upon the transfer completion, the membranes were probed with antibodies against IκBα, phospho-IκBα (Ser32/36), p65/RelA, and phospho-p65/RelA (Ser536) (Cell Signaling Technology). The bands were visualized with Advanced ECL^®^ Western Blotting Detection Reagents (Amersham Biosciences, Amersham, UK). Equal protein loading was checked using an anti-GAPDH antibody (Santa Cruz Biotechnology).

### Mice Experiments

6-week-old male Nc/Nga mice were obtained from SLC Inc. (Shizuoka, Japan) were used for the two set of *in-vivo* experiments as described in Figs 4a and 6a. In the first set, the mice were divided into three groups (n = 5 per group): non-treated control (nt), DNCB-treated only (DNCB-nt) and the topical application of NTP to DNCB-induced mice (DNCB-NTP). After the complete removal of dorsal hairs from the mice, 200 μl of 1% (w/v) DNCB solution (dissolved in a 3:1 [v:v] mixture of olive oil and acetone) and 200 μl of 0.3% (w/v) DNCB solution were treated on dorsal skin as described in Fig. 4a. For NTP treatment, all the mice were anesthetized, but only the DNCB-NTP group mice were exposed to NTP for 5 min. All the mice were sacrificed at a day after the final treatments. In the second set of experiments comparing the effect of NTP with 1% (w/v) HC cream, Nc/Nga mice were divided into 5 groups (nt, DNCB-nt, DNCB-NTP, DNCB-1% HC, and DNCB-NTP-1% HC, n = 5 per group) and treated as described at Fig. 5b. For 1% HC, a conventional drug, 200 mg of LactiCare^®^-HC lotion (GlaxoSmithKline, Brentford, UK) was applied to mouse skin.

### Evaluation of skin dermatitis

The severity of dermatitis was evaluated using the photographs of the mice, those were taken from the first application of the NTP or 1% HC to the end of the experiments. Development of dryness/scarring, edema, erythema/hemorrhage, and erosion/excoriation were scored according to the severity: 0 (none), 1 (mild), 2 (moderate), or 3 (severe). The sum of the 4 individual scores was defined as the dermatitis score^55^.

### Histology

Standard histological PFA fixation, paraffin embedding, and immunostaining protocols were performed. The skin biopsies were fixed in 4% (w/v) paraformaldehyde for 24 h, then embedded in paraffin. The sections (5 μm) were stained with H&E or toluidine blue to monitor histological changes of skin and mast cell recruitment, respectively. Eosinophil peroxidase (EPX) staining was carried out using a goat polyclonal anti–EPX antibody (Santa Cruz Biotechnology) and ABC alkaline phosphatase staining system (Vector Laboratories, Burlingame, CA, USA) with DAB as the staining substrate. The p65/RelA staining within the skin tissue was performed using a rabbit monoclonal anti-p65/RelA antibody (Cell Signaling Technology, Beverly, MA, USA) and an Alexa Fluor^®^ 594 goat anti-rabbit antibody (Invitrogen, Carlsbad, CA, USA). Cell nuclei within the tissues were counterstained using hematoxylin or DAPI.

### ELISA assays

Protein levels of IgE, CCL17, and IFNγ were determined in total protein extracts from 6 mm punch biopsy lesional skin specimens. After measuring the protein concentration of each sample, total protein solutions containing equal amounts of protein were subjected to ELISA assay in duplicate according to the manufacturer’s instructions (IgE and IFNγ: KOMA biotech, Seoul, Korea; CCL17: R&D Systems, Minneapolis, MN, USA). The levels of these cytokines and IgE were normalized to the weight of each tissue specimen and data were presented as relative protein levels.

### Data analysis

Data are presented as the mean ± SD of at least 3 independent experiments. Two-tailed Student’s *t* tests were used to assess statistical significance for differences between means, and the threshold for significance was set at *p* < 0.05.

### Study approval

All experimental protocols for animal care were reviewed and approved by Animal Ethics Committee of Pusan National University (PNU-2013-0265 and PNU-2014-0717) and all animal procedures were performed in accordance with the relevant guidelines.

## Additional Information

**How to cite this article**: Choi, J.-H. *et al.* Inhibition of inflammatory reactions in 2,4-Dinitrochlorobenzene induced Nc/Nga atopic dermatitis mice by non-thermal plasma. *Sci. Rep.* **6**, 27376; doi: 10.1038/srep27376 (2016).
